# Gluten- and casein-free dietary intervention for autism spectrum conditions

**DOI:** 10.3389/fnhum.2012.00344

**Published:** 2013-01-04

**Authors:** Paul Whiteley, Paul Shattock, Ann-Mari Knivsberg, Anders Seim, Karl L. Reichelt, Lynda Todd, Kevin Carr, Malcolm Hooper

**Affiliations:** ^1^ESPA Research, The Robert Luff Laboratory, Unit 133i Business and Innovation CentreSunderland, UK; ^2^National Centre for Reading Education and Research, University of StavangerStavanger, Norway; ^3^Fjellstrand, Norway; ^4^Department of Pediatric Research, Rikshospitalet Medical Centre, University of OsloOslo, Norway

**Keywords:** autism, brain, gastrointestinal, gluten, casein, diet, intervention, intestinal permeability

## Abstract

Dietary intervention as a tool for maintaining and improving physical health and wellbeing is a widely researched and discussed topic. Speculation that diet may similarly affect mental health and wellbeing particularly in cases of psychiatric and behavioral symptomatology opens up various avenues for potentially improving quality of life. We examine evidence suggestive that a gluten-free (GF), casein-free (CF), or gluten- and casein-free diet (GFCF) can ameliorate core and peripheral symptoms and improve developmental outcome in some cases of autism spectrum conditions. Although not wholly affirmative, the majority of published studies indicate statistically significant positive changes to symptom presentation following dietary intervention. In particular, changes to areas of communication, attention, and hyperactivity are detailed, despite the presence of various methodological shortcomings. Specific characteristics of best- and non-responders to intervention have not been fully elucidated; neither has the precise mode of action for any universal effect outside of known individual cases of food-related co-morbidity. With the publication of controlled medium- and long-term group studies of a gluten- and casein-free diet alongside more consolidated biological findings potentially linked to intervention, the appearance of a possible diet-related autism phenotype seems to be emerging supportive of a positive dietary effect in some cases. Further debate on whether such dietary intervention should form part of best practice guidelines for autism spectrum conditions (ASCs) and onward representative of an autism dietary-sensitive enteropathy is warranted.

## Introduction

Pervasive developmental disorders are a complex, lifelong, heterogeneous group of conditions that variably affect the way a person communicates and interacts with people and the environment around them. Autism, Asperger syndrome (AS) and Pervasive Developmental Disorder—Not Otherwise Specified (more commonly known as autism spectrum disorder, ASD) reflect the current primary diagnostic classifications of the condition (World Health Organisation, 1992) although likely to change in revised diagnostic descriptions (Mattila et al., 2011).

The clinical presentation of the autism spectrum conditions (ASCs) as they are becoming known includes primary impairment in areas of: verbal and/or non-verbal communication, the use of reciprocal social interaction (cumulatively known as social affect) and the presence of repetitive or stereotyped behaviors. Various other behaviors may also be present as peripheral features including sensory-perceptual issues (Tomchek and Dunn, 2007) and gait and motor co-ordination problems (Whyatt and Craig, 2012).

The symptoms of ASCs are thought to result from a complex, variable interaction between genetics and environment (Grafodatskaya et al., 2010) though there is currently no genetic or biological test to diagnose the condition. The assessment of an ASC is carried out by detailed observation of overt symptoms combined with an analysis of developmental history according to prescribed criteria.

Contemporary research efforts are being directed away from the search for a condition-specific genetic factor to embrace a more cumulative model based on elevated risk as a function of smaller gene point mutations (Klei et al., 2012) given the heterogeneity present. Included in such a model is the growing realization that the label of autism is not representative of just one condition, but rather the presentation of similar symptoms across various conditions (Novarino et al., 2012). Recent moves to establish specific endophenotypes of ASCs, based on combinations of symptoms and presentation history, influence of co-morbidity, effectiveness of various management strategies, etc., is also gaining popularity (Nordahl et al., 2011).

ASCs are categorized as life-long conditions although there is evidence to suggest differential patterns of development may be present among cases reflective of some diagnostic instability (Fountain et al., 2012). There is a gender disparity in ASCs (Whiteley et al., 2010c). Alongside other developmental conditions (Centers for Disease Control and Prevention, 2010), the numbers of cases being diagnosed has increased in recent years (Autism and Developmental Disabilities Monitoring Network Surveillance Year 2008 Principal Investigators; Centers for Disease Control and Prevention, 2012), thought primarily to be the result of changing diagnostic criteria and better case ascertainment. The role of the environment as part of any real increase in cases has however not been ruled out (Rutter, 2005; Weintraub, 2011) and indeed continues to garner support.

ASCs carry elevated risk for various other comorbid conditions including epilepsy and learning disability (Steffenburg et al., 2003). Such co-morbidities highlight the importance of the brain and neuronal functions to ASCs. Study of these areas has dominated both psychological and neuropsychological theories of aetiology and pathology with a focus on both structural and functional changes to be present.

Various other co-morbidities have been detailed as being over-represented in cases of ASCs. Gastrointestinal (GI) co-morbidities expressing as both functional symptoms and chronic underlying symptoms including coeliac disease (CD) (Barcia et al., 2008; Genuis and Bouchard, 2010) and indications of inflammatory bowel disease-type conditions (Ashwood et al., 2003) have also been reported alongside various nutritional indicators of for example, functional iron deficiency (Latif et al., 2002) in some cases. Although some guidance exists for the systematic inspection of such GI disorders in cases of autism (Buie et al., 2010), continuing discussions on the nature of the GI comorbidity present coupled with ethical concerns on the use of invasive medical procedures in cases of ASCs mean no reliable estimates on population comorbidity presently exist. Importantly a diagnosis of ASC is not currently thought to confer protection against the development of any other health or psychiatric condition over a lifetime.

There is at present no universal intervention for reducing/minimizing the more disabling overt symptoms of ASCs and improving developmental outcomes and quality of life indicators. Society has an important role in the provision of appropriate health, education, and employment opportunities in such a process. Existing best practice guidelines for intervention and management strategies are aimed at ameliorating or managing core and peripheral symptoms based on specialized education and behavioral training (Volkmar et al., 2004). The individual, and their strengths and weaknesses, is an important focus. The emphasis lies in the application of early years intervention aimed at improving developmental outcome where optimal results have, in some cases been suggested to impact on neuronal functioning such as cortical activation (Dawson et al., 2012). Such research is reflective of the perceived plasticity of early development and brain function.

The use of various medications as part of pharmacotherapy is also relatively commonplace for ASCs (Francis, 2005). Such medical intervention provides an important service where comorbid features such as epilepsy are present, but with newer compounds also increasingly looking to address more core features too (Oberman, 2012). At the current time, there is however no single drug or universal medication strategy to treat the condition and its entire range of symptoms.

As with many other cognitive and/or developmentally-defined conditions, specific groups of people with ASCs seem to be at increased risk of various problems associated with eating and diet (Kalyva, 2009). Whether as a consequence of core symptoms based on the variable presentation of inflexible patterns of behavior, issues with fine and gross motor skills or as a result of underlying intolerances to various foodstuffs, several dietary-related issues can be apparent (Martins et al., 2008). Corresponding anthropometric growth measures of people with ASCs have not yet determined any consistent trend as being present as a result of such feeding issues. It has been reported that measures of weight and calculated body mass index (BMI) can present as aberrant in cases of autism (Whiteley et al., 2004; Curtin et al., 2010) seemingly echoing UK and other population trends.

Some people with ASCs have been reported to show an improvement in core and peripheral symptoms following the adoption of specific exclusion diets and/or the variable use of nutritional supplements such as vitamins (Adams et al., 2011), minerals and fatty acids. A diet devoid of gluten (the major protein in wheat, barley and rye) and/or casein (derived from mammalian diary produce) has been one of the more popular interventions suggested to show some effect. This document aims to: (1) summarize the main experimental research carried out on the use of a gluten- and/or casein-free (GFCF) diet for ASCs, (2) summarize the main effects reported following dietary exclusion, (3) highlight the various safety issues associated with dietary use, and (4) discuss the most current theories potentially explanatory of a dietary effect. Although it is beyond the scope of this document to examine all the research conducted on the use of GFCF diets for ASCs, specific studies will be highlighted on the basis of their importance to the research timeline, methodology employed and overall contribution to knowledge.

## Dietary studies: what is the evidence for effect?

Notions regarding the potential for a gluten-free diet (GFD), casein-free diet (CFD), or combined gluten- and casein-free diet (GFCF) to affect the symptoms of ASCs have persisted for many years. Much of the impetus and scientific rationale for the use of such dietary interventions originally stemmed from: (1) models approximating a relationship between food and ASCs with that of dietary related in-born metabolic conditions such as Phenylketonuria (PKU) and (2) dietary investigations suggestive of amelioration of overt symptoms in conditions such as schizophrenia (previously linked to autism) and other psychiatric disorders (Dohan et al., 1969).

The first ever formal description of autistic symptoms contains reference to GI symptoms and dietary issues being present in some cases (Kanner, 1943). Early ideas speculating on a potential link between diet and ASCs were strengthened by some of the writings of Hans Asperger, who provided the initial descriptions of AS, and a suggestion of a relationship between AS and CD (Asperger, 1961). Notwithstanding such potential associations, early research attempting to validate any universal link between ASCs and CD were in the most part unsuccessful (Pavone et al., 1997) although retaining the possibility of a connection between a proportion of cases of ASC and co-morbidity of CD (Barcia et al., 2008; Genuis and Bouchard, 2010). Contemporary use of a diet devoid of gluten and/or casein for ASCs is now considered to be widespread; despite no formal published guidelines yet accepting dietary intervention as a viable intervention strategy for the condition.

Meta-analyses of the specific findings of the various trials of such dietary intervention for ASCs published in the peer-reviewed scientific literature have been summarized by several authors (Knivsberg et al. 2001; Mulloy et al., 2010, 2011) including the Cochrane Library of Systematic Reviews (Millward et al., 2008). The main conclusions from such meta-analyses suggest caution in the universal adoption of GFCF dietary intervention for ASCs whilst stressing the need for further controlled research to ascertain any significant effect. A thorough examination of the individual evidence included in these texts is beyond the scope of this document. Several pertinent and additional studies published after the Cochrane review (post-2008) do, however, necessitate further description.

In the early 1990s Knivsberg, Reichelt and colleagues based at various sites in Norway published initial and follow-up behavioral and psychometric data for a small group of people (*n* = 15) with ASCs on a GFCF diet (Knivsberg et al., 1990, 1995). For many, these studies were the first primary evidence for the potential effectiveness of a GFCF diet for ASCs adding scientific validity to the array of anecdotal observations previously described, and strengthened by the long period of dietary exclusion between publications. The downside to these initial studies lay predominantly with the open, non-randomized methodology employed together with a lack of suitable blinding; thus introducing potential bias into the interpretation of results obtained.

The Norwegian team have subsequently been involved in further experimental studies of GFCF dietary intervention for ASCs. Two of these studies (Knivsberg et al., 2002; Whiteley et al., 2010a) were randomized controlled trials (RCTs) lasting for 1 and 2 years respectively. Both studies indicated significant positive group effects on several measures of behavior and development indicative of potential improvements to symptoms for some children with ASCs on diet.

One of these RCTs (ClinicalTrials.gov NCT00614198) published in 2010 (Whiteley et al., 2010a) was not included in the most recent Cochrane Library Review (Millward et al., 2008) given its publication date. This study known as “ScanBrit” used an adaptive study design responsive to intermediate analysis of results (a “drop-the-loser” design) to analyze any dietary effect (*n* = 72). The main findings indicated statistically significant changes to both core and peripheral behaviors in the diet group in the first 12 months of study followed by indications of a plateau effect of diet following 12 months further study. Results also indicated a substantial degree of variability in individual response to intervention.

Another recent single-blind investigation of potential dietary effect (Johnson et al., 2011) (*n* = 22) reported no overall difference between diet and non-diet groups following 3 months of study. This despite finding some gains in areas previously described by Whiteley et al. (1999, 2010a) and others related to dietary intervention. Anecdotal but numerous clinical observations leading up to the formal studies of Reichelt and Knivsberg (Knivsberg et al., 1990, 1995) indicated that GFCF dietary intervention needed to be implemented for at least 6 months before one could reasonably assess response or not and why the subsequent ScanBrit trial (Whiteley et al., 2010a) used a considerably longer implementation period.

Double-blind RCTs of a GFCF or individual CFD or GFD intervention for ASCs are currently few in number; primarily as a result of the cost involved to perform such a study and issues on how to ensure a double-blind methodology is implemented and adhered to. One group (Elder et al., 2006) has reported results from a double-blind trial; another group reported double-blind results following dietary challenge (Lucarelli et al., 1995). A further trial is cited as on-going (Diet, and behaviour in young children with autism, 2012), but at the time of writing has not been published in the peer-reviewed literature. The results from Elder et al. (2006) suggested no significant group effects as a result of dietary intervention in place. Whilst methodologically sound, this trial has however been criticized over the small participant group (*n* = 15) used, measures of dietary adherence and the short study period (6 weeks on diet and 6 weeks of no diet). The trial by Lucarelli et al. (1995) contained a double-blind element during dietary challenge and is one of two trials where investigations into whether a GFD or a CFD alone may have any effect for people with ASCs were carried out. Lucarelli et al. examined the effects of a CFD (*n* = 36). They reported an improvement in group behavior scores of autistic behaviors after 8 weeks of intervention. They also reported a worsening of autistic symptoms when a casein-challenge was introduced. Whiteley et al. (1999) measured response to a GFD alone over a period of 5 months during an open trial (*n* = 22). Results were slightly less clear in this study despite some indications of significant improvements to autistic symptoms in specific participants. Again, variability in response to intervention was reported amongst the participant group.

On the basis of these and other smaller trials, the experimental research base examining the use of a GFCF diet for ASCs can most accurately be described as mixed yet broadly suggestive of decreased autistic symptoms and improved developmental outcome for some individuals. These findings are complemented by other more survey-based research (Pennesi and Klein, 2012). The main caveat being that methodological issues associated with various forms of bias still persist to potentially confound experimental results. Such biases include: a lack of placebo conditions in trials, small participant numbers, short trial duration, problems associated with the outcome measures used and problems with the monitoring of dietary adherence.

An additional important issue not yet adequately covered by many of the dietary studies completed so far relates to the measurement of clinical *vs*. statistical significance; that is analyzing day-to-day performance of individuals on diet and determining what (if any) positive changes are present that increase quality of life and overall daily living and functioning for individuals rather than just providing statistical evidence of effect.

## What kind of effects are observed?

At the time of writing, there is no universal consensus on the type of effects experimentally observed following successful outcome from a GFCF diet for ASCs. Taking the various studies of diet into account, reported positive effects can be broadly categorized into several areas to include core autism and peripheral symptoms:

– Communication and use of language (Knivsberg et al., 1990, 1995, 2002; Lucarelli et al., 1995; Whiteley et al., 1999, 2010a; Johnson et al., 2011).
– Attention and concentration (Knivsberg et al., 1990, 1995, 2002; Lucarelli et al., 1995; Whiteley et al., 1999, 2010a).
– Social integration and interaction (Knivsberg et al., 1990, 1995, 2002; Whiteley et al., 1999, 2010a).
– Self-injurious behaviour/altered pain perception (Knivsberg et al., 1990, 1995; Lucarelli et al., 1995; Whiteley et al., 1999).
– Repetitive or stereotyped patterns of behaviour (Knivsberg et al., 1990, 1995; Knivsberg et al., 2002).
– Motor co-ordination (Knivsberg et al., 1990, 1995; Whiteley et al., 1999).
– Hyperactivity (Whiteley et al., 2010a; Johnson et al., 2011).

There have also been suggestions of potential variable abatement of co-morbid conditions such as epilepsy and seizure-type disorders (Knivsberg et al., 1990, 1995) following dietary use for ASCs and coincidental seizure activity following reinstallation of a gluten-load (Whiteley et al., 1999). Similar case studies describing a reduction of seizure activity have also been reported in CD following use of a GFD (Pratesi et al., 2003). Changes to anti-epileptic or other medication as a result of the introduction of such dietary intervention for ASCs have not been advocated without consultation with the supervising medical physician. Whether such dietary intervention represents an alternative treatment modality for some forms of epilepsy independent of ASC co-morbidity has also not been investigated. The use of a ketogenic diet in respect to specific types of treatment resistant epilepsy (Lee and Kossoff, 2011) and also autism (Evangeliou et al., 2003) may potentially offer some clue to effect given the likely overlap between dietary regimes.

Within the spectrum of cases of ASCs, anecdotal reports of responders and non-responders to dietary intervention persist, although no universal criteria to account for response differences has yet been formulated. Given the heterogeneous spectral nature of ASCs, it is highly unlikely that everyone will benefit from such a dietary change.

Chronological age is thought to be a factor in response. Indeed, the experimental studies conducted thus far have predominantly looked at dietary response in children and young adults with ASC. Effects are thought to be similar to the ethos behind other more educationally and behaviorally-based interventions, where younger children are reported to show more pronounced effects from diet. Whether this is due to plasticity and maturational factors in brain function for example or purely coincidental as a function of known diagnostic instability at younger ages (Charman et al., 2005) is unknown at the current time.

Anecdotal reports of improvements to some of the symptoms of ASCs following introduction of a GFCF diet where functional bowel problems (diarrhoea, constipation, alternating stools) have emerged. There is some evidence to corroborate a potential connection between ingestion of specific dietary components such as dairy products and the presence of functional GI problems in ASCs (Afzal et al., 2003). Further investigations are however required, and indeed on-going, into whether this forms universal criteria for positive response to diet (ClinicalTrials.gov NCT01116388) (A study to assess the role of a gluten free-dairy free (GFCF) diet in the dietary management of autism associated gastrointestinal disorders, 2012).

## Risks and safety issues

The use of a GFCF diet for ASCs carries a number of potential risks. Current, best evidence suggests that whilst the effects of dietary intervention may largely be apparent during the first year of intervention (Whiteley et al., 2010a), there appears to be a continued requirement for the diet to be in place for much longer assuming initial positive effects are witnessed (Knivsberg et al., 1995).

Whilst potential nutritional deficiencies in ASCs are a major cause for concern (Arnold et al., 2003)—for example, calcium intake following the exclusion of dairy products—the limited investigations completed so far suggest that with suitable support, dietary intake need not be adversely affected by introducing such a diet (Cornish, 2002; Adams et al., 2008). The increasing range and availability of GFCF foods may help alleviate the feeding problems described in ASCs based on limited product range and other personal preferences (taste, texture, etc.). Further investigations are however, required on the basis of nutritional value and fat, protein and sugar content of such alternative foods (Mariani et al., 1998) specifically where anthropometric measures of dieters may already be irregular.

Anthropometric information following GFCF dietary use in ASCs is sparse. Allowing for geographical and ethnic differences, case study reports suggest a trend toward normalization of growth parameters following dietary intervention (Hsu et al., 2009) although no large scale studies have yet been conducted.

Pathology following the use of a GFCF diet has been suggested; specifically related to bone health and use of a CF diet in ASCs (Hediger et al., 2008). There is continuing debate as to whether this is due to specific deficiencies as a function of dietary exclusion, a consequence of abnormal eating patterns in ASC generally or part of a broader physiological problem with the absorption of nutrients associated with the condition (Clark et al., 1993; Stewart and Latif, 2008; Herndon et al., 2009) particularly where bowel or malabsorptive issues may already be present. Accompanying issues with functional levels of important vitamins linked to calcium homeostasis such as vitamin D have also been identified (Neumeyer et al., 2012).

Whilst not specifically a safety issue of the GFCF diet, the use of various nutritional supplements as part of the dietary regime alongside dietary exclusion also requires comment. Children following a GFCF diet are perhaps more likely to be also following other complementary and medicine (CAM) approaches at the same time as their diet, particularly when GI comorbidity is also apparent (Perrin et al., 2012). Bearing in mind the often intricate balance required between specific vitamins and minerals (e.g., calcium supplementation affecting iron absorption; Cook et al., 1991), professionals have been advised to be mindful of such adjunctive interventions.

Finally, as with any potential intervention for ASC, great thought is required into the “necessity” of such an intervention and the likely cost/benefit ratio to individual users given the current lack of formal best-responder data. Unlike more traditional conditions where such dietary interventions are employed, people with ASCs may not be able to readily understand why such a diet is being used or communicate any preference on its application or not. Indeed, food and established feeding patterns may be a great source of comfort, stability, routine and coping to some; use of a GFCF diet may likely upset some people with ASCs especially during the early days of intervention. In such cases, great care is required to involve all persons potentially affected by such dietary changes (person, family, school, support services, etc.) to ensure appropriate monitoring with regards to effectiveness and safety; also potentially including observations on dietary compliance.

## Potential modes of action

At the current time, no universal theory has been accepted to account for the effect (or non-effect) of GFCF dietary intervention on behaviour and development in ASCs. Given the heterogeneity observed in the presentation of overt symptoms in ASCs, it is likely that more than one model of dietary effect may pertain in different cases. Whiteley et al. (2010b) summarized the main hypotheses commonly ascribed to dietary success/non-success. As per previously, ASCs are not thought to be protective of co-morbid conditions that may have a dietary link where for example, low levels of co-morbid PKU and ASC have been reported (Baieli et al., 2003).

Indeed, an early analogy with PKU had been put forward (Seim and Reichelt, 1995) in relation to GFCF diets focusing on the cumulative effects of protein and peptide aggregates crossing the blood-brain barrier to exert a neuronal action, stressing a collective, chronic effect rather than an acute action.

The possibility of an underlying metabolic condition being connected to dietary response has been further extended (Shattock and Whiteley, 2002) from conditions such as schizophrenia (Dohan et al., 1969). The theory suggests that abnormal porosity of the intestinal wall (gut hyperpermeability or leaky gut) and potentially other membranes throughout the body, combines with inadequate hydrolysis of dietary proteins to produce onward effects to the central nervous system (CNS).

Some support for the model has been published; specifically preliminary indications of peptiduria (Reichelt et al., 2012) appearing in cases of autism coinciding with antibody production to peptides (Vojdani et al., 2004) and the effects of administration of specific dietary-derived peptides on behavior (Sun and Cade, 1999) and neuronal functioning (Sun et al., 1999) in animal models. A role for incomplete elimination of bovine-derived peptides impacting on psychomotor development and autism has also been reported (Kost et al., 2009).

Alongside, gut hyperpermeability has been reported in approximately a quarter to a third of children with an ASC examined (D'Eufemia et al., 1996; Boukthir et al., 2010; de Magistris et al., 2010) although not universally so in all investigations (Robertson et al., 2008) complemented by findings in other, more GI-related conditions (Cummins et al., 1991). Importantly also, there are indications of a reduction of GI permeability in those cases where a GFCF diet has been implemented in cases of autism (de Magistris et al., 2010) similar to processes described in CD (Cummins et al., 1991). This point in particular may also account for the findings reported by Robertson et al. (2008) of no abnormal permeability in their cohort, who crucially included participants already following a special diet at the time of sampling. A role for inflammation and inflammatory signaling and processes similar to those described in cases of schizophrenia (Severance et al., 2012a) requires further investigation as do potential issues governing the integrity of the intestinal barrier via sulphonation (Bowling et al., 2012), tight-junction modulators (Fasano, 2012) and any contributing role for pathogenic agents (Severance et al., 2012b).

The gut-brain model in its entirety however has not yet been fully validated, specifically with regards continuing dispute on the detection of dietary-derived peptides in biological fluids as evidence of abnormal protein metabolism (Cass et al., 2008). There is preliminary evidence suggestive of potentially relevant compounds present in urine correlating with suggested best responder characteristics (Wang et al., 2009) although further investigations are warranted. The implications of such findings for screening and recommendations of potential dietary effectiveness are therefore the source of continuing debate.

Focus has also shifted to more fundamental problems with carbohydrate metabolism as potentially being implicated in a dietary effect. Williams et al. (2011) reported on decreased mRNA expression for disacharidases and hexose transporters present in cases of ASCs. This follows on from earlier research hinting at reduced dissacharidase activity (Kushak et al., 2011) potentially indicative of underlying lactose intolerance to be present. Combined with a suggestion of some involvement for the composition of GI bacterial species in cases of ASCs (Parracho et al., 2005; Clayton, 2012) and possible effects from bacterial translocation, this remains an area in need of further investigation.

Various individual accounts of CD and ASCs have been documented (Barcia et al., 2008; Genuis and Bouchard, 2010). Genuis and Bouchard (2010) detailed the rapid resolution of GI symptoms and corresponding abatement of autistic symptoms following implementation of a GFD. Similar case reports have been highlighted with regards to schizophrenia and overlapping CD, together with documented brain imaging changes (De Santis et al., 1997). Likewise indications of specific allergies to foods such as gluten and casein in some people with ASCs have also been highlighted (Lucarelli et al., 1995; Jyonouchi et al., 2002). Additional studies incorporating the exclusion of dietary gluten and casein in related conditions such as attention-deficit hyperactivity disorder (ADHD) have also noted positive effects on symptoms (Pelsser et al., 2011) particularly in cases of overlapping CD (Niederhofer, 2011) where both somatic and psychological presentation were affected. Combined however, such co-morbidities are not thought to be able to account for all cases of success despite no commonplace screening for such potential issues in ASCs and the possibility of non-CD mediated sensitivities (Biesiekierski et al., 2011).

## Conclusions

Experimental studies on the use of a GFD, CFD, or combinatorial GFCF diet for ASCs have suggested an amelioration of symptoms and improved developmental outcome for at least a proportion of people on the autistic spectrum. That being said, various methodological issues potentially biasing results remain which, combined with a lack of generalizable information on mode of action and best-responder data, have limited the impact of such findings over the years.

More recent controlled longitudinal studies examining group dietary effectiveness alongside an increasing recognition of individual cases of food-related co-morbidity and evidence of more consolidated biological mechanisms potentially at work, offer a favorable evidence base for at least a partial effect of diet on some cases of ASCs.

There is a continued requirement for further study on the potential role of dietary intervention for ASCs. Future controlled trials including blinded and placebo elements are necessary carrying appropriate power of study by sample size and duration. Based on the significant heterogeneity present in ASCs and the likelihood of various “autisms” manifesting similar presentation, further thought should also be given to the concept of best- and non-responders to this type of intervention. So for example, (1) screening for GI and/or potentially relevant pathogenic comorbidity, (2) measuring gut hyperpermeability, (3) examining gut microbial populations and food-related enzyme activities, and (4) ascertaining the presence of inflammatory processes, either peripherally in GI tissue or more centrally, might all be included as parameters for future dietary investigations. Similarly, measuring any relationship between behavior and GI function over the course of dietary intervention may offer some information about any connection between these factors.

Given the evidence hinting at neurological changes following the implementation of dietary intervention in related conditions, future research might also benefit from looking at brain structural and biochemical changes in cases of ASCs adopting dietary intervention. Indeed, the gut-brain relationship, seemingly so important to explaining the role of dietary intervention in best-responder cases, is a woefully under-researched area with ASCs in mind.

Finally but perhaps just as important, is a need to focus on the measurement of clinical changes to symptoms alongside statistical changes to psychometric or other assessment tools in view of the restrictiveness of the dietary regime. This point in particular reflects the fact that not everyone who might potentially benefit from dietary intervention will necessarily be able to implement such a restrictive regime, or indeed, want to.

The growing emphasis on various phenotypes for ASCs provides a template for conceptual changes to the way ASCs are viewed; where a ‘diet-related autism phenotype’ may be a target for future research and indeed a marker for efficacy of dietary intervention. Further discussions on whether such dietary intervention should form part of best practice guidelines for ASCs and onward representative of an autism dietary-sensitive enteropathy is warranted.

## Conflict of interest statement

Kevin Carr, Malcolm Hooper, and Paul Whiteley are directors of ESPA Research, a UK subsidiary organization which carries out research into ASCs including investigations on the use of a gluten- and casein-free diet as an intervention for autism and related conditions. Lynda Todd is an employee of ESPA Research. Paul Shattock is Chairman of ESPA (Education and Services for People with Autism) and has a son with autism. Malcolm Hooper is also a Trustee of ESPA. Kevin Carr, Paul Shattock, and Paul Whiteley are directors and shareholders of Analutos Ltd. in the UK which provides mass spectrometric and other analytical services to various sectors of the healthcare, chemical and pharmaceutical industries. Karl Ludwig Reichelt is an unpaid consultant to Biomedical Laboratory in Norway providing mass spectrometric and other analytical services to various healthcare industries. Ann-Mari Knivsberg and Anders Seim declare that the research was conducted in the absence of any commercial or financial relationships that could be construed as a potential conflict of interest.

## Abbreviations

ADHD: Attention-deficit hyperactivity disorder
AS: asperger syndrome
ASC: autism spectrum condition
ASD: autism spectrum disorder
CD: coeliac disease
CF: casein-free
CNS: central nervous system
GFCF: gluten-free, casein-free
GFD: gluten-free diet
GI: gastrointestinal
PKU: phenylketonuria
RCT: randomized controlled trial.
